# The Choreography of Group Affiliation

**DOI:** 10.1111/tops.12320

**Published:** 2018-01-12

**Authors:** Jorina von Zimmermann, Staci Vicary, Matthias Sperling, Guido Orgs, Daniel C. Richardson

**Affiliations:** ^1^ Department of Experimental Psychology University College London; ^2^ Department of Psychology, Goldsmiths University of London; ^3^ Independent Artist and Choreographer London United Kingdom

**Keywords:** Synchrony, Coordination, Group behavior, Pro‐sociality, Affiliation

## Abstract

When two people move in synchrony, they become more social. Yet it is not clear how this effect scales up to larger numbers of people. Does a group need to move *in unison* to affiliate, in what we term *unitary synchrony*; or does affiliation arise from *distributed coordination,* patterns of coupled movements between individual members of a group? We developed choreographic tasks that manipulated movement synchrony without explicitly instructing groups to move in unison. Wrist accelerometers measured group movement dynamics and we applied cross‐recurrence analysis to distinguish the temporal features of emergent unitary synchrony (simultaneous movement) and distributed coordination (coupled movement). Participants’ unitary synchrony did not predict pro‐social behavior, but their distributed coordination predicted how much they liked each other, how they felt toward their group, and how much they conformed to each other's opinions. The choreography of affiliation arises from distributed coordination of group movement dynamics.

## Abstract 2

Dance provides natural conditions for studying relationships between coordination patterns and human experience. von Zimmermann and colleagues investigate whether relatively simple or more complex forms of movement coordination are related to pro‐social experiences during group dance. They find that pro‐social experience depends on the degree to which movement patterns are distributed and diversified, but not the degree to which movement patterns are simply unitary.

## 1 Introduction

Coordinated movement pervades social life (McNeill, 1995). A group of people on a night out, for example, could be coordinated as they dance together at a concert, as they go for a walk together talking, and as they ride the train home, swaying from side to side. Their motion will be intentionally synchronized to other audience members and the rhythm of the music in the first case; their footsteps loosely coordinated between chatting couples in the second; and their body sway tightly bound, although unintentionally so, to other commuters and the movement of the train in the last.

Psychologists have shown that between pairs of people, such bodily coordination produces feelings of liking and affiliation (Chartrand & Bargh, 1999; Marsh, Richardson, & Schmidt, 2009; Oullier, de Guzman, Jantzen, Lagarde, & Kelso, 2008; Schmidt & Richardson, 2008), and there is emerging evidence that also larger numbers of people moving together become more pro‐social (Codrons, Bernardi, Vandoni, & Bernardi, 2014; Reddish, Fischer, & Bulbulia, 2013; Tarr, Launay, Cohen, & Dunbar, 2015). Yet the specific characteristics of group movement dynamics that induce the pro‐social effects of “moving together” remain unclear. Do pro‐social effects require that all members of a group move in unison at the same time as each other, and intentionally so, or simply that there is some similarity in the movement dynamics that emerge across the group? What, in other words, is the choreography of group affiliation?

In the imitation and coordination literature it has been found that actions that are similar in form and timing enhance cooperation and rapport (Fischer, Callander, Reddish, & Bulbulia, 2013; Lumsden, Miles, & Macrae, 2014). Indeed, the evidence from multiple pair studies suggests that coordinated physical action can function as “social glue” that binds people together (Valdesolo, Ouyang, & DeSteno, 2010), increasing liking (Hove & Risen, 2009; Launay, Dean, & Bailes, 2014), perceived and experienced feelings of togetherness and similarity (Lakens, 2010; Lumsden et al., 2014), cooperation (Wiltermuth & Heath, 2009), and conformity between interaction partners (Dong, Dai, & Wyer, 2015).

There are two possible ways that these phenomena of dyadic behavior could scale up to large groups of people. The first is that a synchronous group is like one enlarged dyad, in which all members are moving in unison, in time with each other. We term this *unitary synchrony*, and it might be seen in groups that are dancing or marching to a common rhythm. The second possibility is that a synchronous group is like many synchronized dyads in which a network of coupled members are moving in time with each other. In this case, a group's coordination consists of patterns of movement that re‐occur among its members but are not performed by the whole group at the same single point in time. We term this *distributed coordination*, and it might be seen in crowds walking on the street or at a football stadium. There is no “conductor” in this form of coordination to which all members are aligned, but across the group, coordination emerges from multiple couplings between its members, without any external signal. Both *unitary synchrony* and *distributed coordination* are logical extensions of synchronous phenomena in dyads and both can be observed in group behavior. However, from the current literature, it is not clear which type of coordination leads to affiliation and enhances pro‐sociality in a group.

Most studies on behavioral coordination actively enforce *unitary synchrony* by explicitly instructing it or by providing a rhythmical external signal to which movements are temporally aligned. For instance, groups with a shared goal of synchronizing their movements or speech to a metronome beat act more cooperatively than groups that are either prevented from moving or speaking in synchrony or for which shared intentionality is not given (Reddish et al., 2013). The authors argue that groups, which succeed in intentional synchronization, construe their behavior as successful cooperation, thereby reinforcing pro‐social and cooperative tendencies. Similarly, synchronized dance movements in small groups were found to significantly increase ingroup pro‐sociality ratings in comparison to groups, which performed different movements to the same piece of music (Tarr et al., 2015). In both of these studies, an external signal conducts group performance, effectively reinforcing group movement as one by providing a common rhythm that sustains synchrony. This setup differs substantially from those real‐life situations in which behavioral coordination emerges spontaneously, unintentionally, and without external guidance such as when commuters are navigating their way through the tube system. Applying principles from dance and choreography (Vicary, Sperling, von Zimmermann, Richardson, & Orgs, 2017), we were interested in studying such emergent behavioral coordination, in the absence of explicit instructions to synchronize, or an external signal guiding movement.

We developed a movement workshop for small groups, led by one of the authors (MS), a professional choreographer. Dance and choreography are useful tools to study social interactions since they provide a naturalistic, yet highly controlled setting (Christensen & Jola, 2015; Cross, Acquah, & Ramsey, 2014; Orgs, Caspersen, & Haggard, 2016; Sevdalis & Keller, 2011). In fact, dance and music may have specifically evolved to promote social bonding in societies (Dunbar, 2012; Dunbar, Kaskatis, MacDonald, & Barra, 2012; Savage, Brown, Sakai, & Currie, 2015). In our experiment, groups of participants performed a set of choreographic movement tasks, designed to lead them to move in either synchrony or asynchrony in the absence of explicit instructions. Concurrently, wrist accelerometers provided online measurements of group movement dynamics. After the workshop, every group took part in a behavioral testing session. Group members gave ratings of each other and their feelings toward their group and reported their agreement to a selection of opinion statements by standing along a giant Likert scale on the floor. Since they could see each other's movements as they made their choice, we quantified their physical proximity as an index of their conformity, or the degree to which they were influenced by each other.

Our aim was to assess how these measures of affiliation and conformity are predicted by group movement dynamics. Specifically, we performed cross‐recurrence quantification analysis (Coco & Rick, 2014) on participants’ collective movements to quantify the temporal coupling between pairs of individuals, and averaged across those measures to quantify group coordination. Cross‐recurrence analysis allowed us, first, to quantify the degree to which participants were accelerating at the same time as each other, known as the simultaneous recurrent rate (SRR). This provided us with a measure of *unitary synchrony*. Secondly, cross‐recurrence analysis provided a measure known as determinism (DET), which quantified the degree to which participants engaged in similar movement sequences with each other that could be simultaneous or not. For example, if one person made a distinctive hand gesture, and a few moments later, another copied it, that would be reflected in higher DET. In this way, DET becomes a measure of *distributed coordination* within groups. Cross‐recurrence analysis, therefore, allows us to dissociate different forms of synchrony based on the temporal features of collective movements (Brick & Boker, 2011) and to isolate the respective contribution of these different forms of synchrony to affiliation.

By manipulating movement synchronization implicitly through choreographic exercises and measuring the distinct temporal features of *unitary synchrony* and *distributed coordination* across individuals, we can test two predictions about the relationship between group movement and affiliation: One hypothesis is that pro‐social behavior in groups will result primarily from unitary synchrony, the prolonged simultaneous movements of all group members (Reddish et al., 2013; Tarr et al., 2015). An alternative hypothesis is that higher levels of group affiliation and conformity will be produced by distributed coordination, the increased movement coupling between group members. To the best of our knowledge, this study is the first to assess these two possible routes to heightened levels of group affiliation simultaneously.

## 2 Methods

### 2.1 Participants

Eighty adults volunteered to participate in the experiment (*M*_age_ = 27.3, *SD*_age_ = 10.52, number of males = 24), which was run in a professional dance studio that we were able to access over the course of one weekend. This achieved our goal to recruit a number of participants that was comparable to other group movement experiments (e.g., Reddish et al., 2013; Wiltermuth & Heath, 2009). An overwhelming majority of participants (86%) reported zero to very little (years < 2) dance experience, with the remaining 14% reporting an average of 5.8 years professional dance performance or training.

Since we were drawing from a local participant pool, we were also concerned that some participants might be familiar with each other. However, reported levels of familiarity did not differ between our groups. On a scale of 1 (unknown) to 3 (familiar) participants rated their familiarity to each individual in their group. The mean ratings were 1.13 (asynchronous condition) and 1.14 (synchronous condition). The participants were informed that this was a research on the “effects of exercise on brain function” and were unaware of the true research hypothesis until after the experiment was complete. All participants were paid £20 in cash for their participation.

### 2.2 Ethics statement

Ethical approval was obtained from School of Social Sciences Research Ethics Committee, Brunel University. All participants provided written informed consent before the beginning of the study.

### 2.3 Procedure

Ten groups completed the experiment, five groups for each movement condition (synchrony vs. asynchrony). Prior to the experiment, all participants received a pre‐activity questionnaire, asking for demographic information, dance experience, and personality measures. The experiment consisted of two phases, a movement workshop and a psychological testing session, and was conducted over the course of 2 days. Each experimental session took around 60 min. In the movement workshop, participants were run in groups of 5–12, either in the synchronous or asynchronous condition. The condition order was partially randomized to obtain close to even numbers of participants across conditions (synchronous = 38, asynchronous = 42). The experiment was double blind, so that the researchers conducting the psychological tests were unaware of whether the participants had moved in synchrony or in asynchrony.

#### 2.3.1 Movement workshop

Participants entered the performance space together, and they were assigned a numbered bib and given an Empatica E4 wrist sensor that recorded acceleration in three dimensions at 32 hz (Garbarino, Lai, Bender, Picard, & Tognetti, 2014). One of the experimenters also wore a wrist sensor and used the single “event marker” button on this sensor to indicate the start and end of the overall movement session and of each individual task during the workshop.

A professional choreographer then introduced participants to three movement tasks. These focused on movements of walking/running in circles, falling/tipping into walking, and arm swinging, always performed in this order. The tasks were preceded and followed by a brief warm–up and cooling–down session. In contrast to what is commonly perceived as conventional practice in dance, our choreographic tasks did not ask participants to memorize and replicate a fixed routine of movements to music or a common rhythm. Rather, the tasks required participants to flexibly apply familiar movements within a clearly defined rule structure, but in the absence of a guiding signal or continuous instructions. In the synchrony condition, instead of establishing an explicit goal to move in unison, the successful completion of our tasks *implied* movement synchronization, without any guidance to how it could be achieved. Importantly, following standardized explanations of the task rules and an initial practice period, participants performed the tasks without any feedback by the choreographer or any other external guiding signal. Movement tasks comprised a defined movement vocabulary and were either performed together as a group (synchrony condition), or participants were instructed to explore movement options on their own (asynchrony condition). Task instructions made no explicit references to group behavior or coordination, but solely focused on the spatial and temporal characteristics of the movement vocabulary. For example, participants were either asked to move in one circle (synchrony condition) or multiple circles (asynchrony condition). The task instructions of the circling task are provided in the supplementary material as an example (Section A). We also provide a more detailed description and discussion of these tasks in the context of dance aesthetics in Vicary et al. (2017), including video examples.

##### 2.3.1.1 Circling task

Participants were instructed to visualize a circle drawn onto the floor and to continually move around the edge of this imaginary circle, in varying velocity, direction, or location, and which could increase or decrease in size. In the *asynchronous* condition, participants were asked to find a space on the floor and to imagine their own circles. This meant that participants would change the size, speed, direction, and location of their circle irrespective to the characteristics of the circles made by the rest of the group. In the *synchronous* condition, participants were placed into one communal circle and instructed to perform the task while maintaining this shared circle.

##### 2.3.1.2 Falling task

Standing participants were instructed to imagine a laser light pointing in a straight line from the top of their head to a point on the ceiling directly above them. Participants were then asked to gradually shift this imagined point of light forwards on the ceiling, by slowly tilting their whole vertical axis forwards, plank‐like, until they momentarily lost their balance and experienced a split second of being in free fall. At this moment of “falling”, the participant was to catch themselves by allowing their feet to move forward and walking out of the falling movement to a new location in the room. Once in a new location, the participant was to repeat the falling movement. In the *asynchronous* condition, the participants were asked to explore different timings of the fall and to vary the time in between falling movements. In the *synchronous* condition, the participants were asked to “have one fall in the room,” which required participants to coordinate the timing of their individual falls.

##### 2.3.1.3 Swinging task

**Figure 1:**
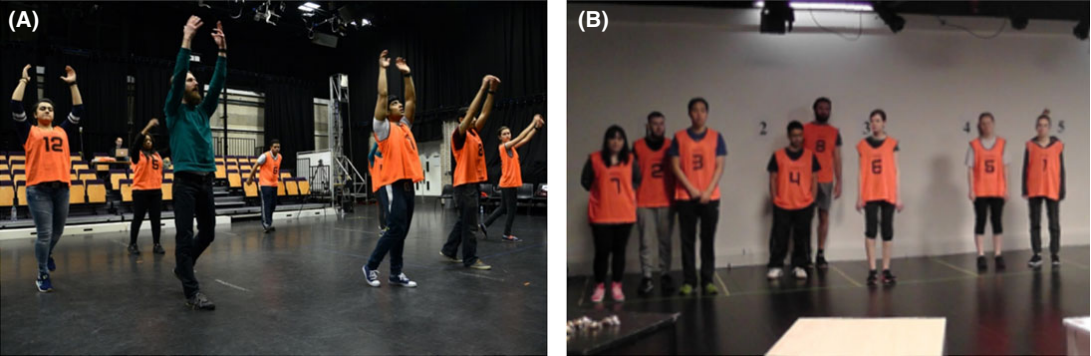
(A) The swinging task in synchrony condition of the movement workshop. (B) The opinion task in the behavioral testing session.

In the Swinging task, participants were instructed to shift their weight back and forth while swinging both arms up and down (with arms up to the front as weight was shifted forward). This movement was repeated continuously and participants were asked to gradually shift the direction and the speed of swinging. In the *asynchronous* condition, participants performed this task on their own. In the *synchronous* condition, participants stood in a circle facing the same way (see Fig. 1).

#### 2.3.2 Behavioral testing session

After the movement workshop, participants were led to a different room with different experimenters, who were unaware of the movement condition they had been in. Participants individually completed questionnaires and then engaged in an opinion task, designed to measure their levels of conformity. Participants also engaged in group cooperation tasks, which we describe and analyze in a separate forthcoming paper. Here, we focus exclusively on measures of individual liking, group affiliation, and conformity. The order of the tasks was the same for each group.

##### 2.3.2.1 Rating task

Participants first sat in a circle and each filled out a post‐activity questionnaire on a tablet of the brand ASUS. They rated their experience of the workshop (14 items, 7 items reverse coded), and their group (16 items, 7 reverse coded). For the group ratings, we adapted nine items designed to measure entitativity and rapport from Lakens and Stel (2011), and reverse coded seven of these items. We also presented participants with a scale to measure self‐group overlap (Schubert & Otten, 2002). Referring to the numbers on their bibs, they rated each group member's performance and how much they liked them individually as well.

##### 2.3.2.2 Opinion task

Participants gave their opinions to survey items by moving around a 15 m Likert scale we had drawn on the floor. We marked out 7 regions along one end of the room, and labeled them with numbers. Participants began each rating by standing in the middle of the room (see Fig. 1b). The experimenter then read out statements such as “capital punishment is morally justifiable,” and participants walked to the regions on the floor to signal their response, from 1 (false) to 7 (true). Every time before the participants moved back to the middle of the room, they were photographed and their positions later recorded by bib number.

## 3 Data processing

### 3.1 Movement workshop

Before the workshop began, the acceleration signals of all sensors were temporally aligned by moving them up and down together. In pre‐processing, each series was aligned with these reference points and then segmented into the three tasks, using the time‐markers recorded by the researcher during the experiment. The magnitude of acceleration was calculated from the three‐axis acceleration data by taking the square root of the sum of squared x, y, and z values, leaving one time series vector for each participant The data were checked for obvious errors or outliers (i.e., sensor failure) and was de‐trended by removing the mean. Since performing the movement task required some practice, only the final 4 min of each task were then extracted for further analysis.

Cross‐recurrence quantification analysis (CRQA) was used to quantify the temporal coupling between the movements of individuals and across groups in our workshop (crq R package, Coco & Rick, 2014). Points of recurrence are defined as moments in time when series are in the same state, with some threshold. A full cross‐recurrence consists of the recurrence between time series, aligned at all possible lag values. The recurrence rate is simply the percentage of points of recurrence across all possible lags. It represents the overall similarity between time series, in this case, similarity of the movement acceleration profiles of our participants. The SRR is the proportion of time that series are in the same state, when they are aligned at exactly the same time. It shows the degree of direct, moment‐by‐moment synchrony between two time series, as an index of *unitary synchrony*. Also, we computed determinism (DET) as the proportion of time that time series are recurrent in extended sequences, at any lag alignment. Although points of recurrence are calculated moment‐by‐moment, DET requires that two sequences have similar states over the course of several samples. In our data, this corresponds to the degree to which two people are engaged in movement trajectories of around 1–2 s in duration that are similar to each other but can be non‐simultaneous. In this way, DET is how we operationalize *distributed coordination*.

Since CRQA is a *pairwise* method, measures were calculated for every possible pair within every group. We then averaged over these pairs of individual and group measures. The degree to which an individual was coupled with their group was given by averaging across their pairwise recurrence with all their other group members. The recurrence in a group was given by averaging across all individuals within it (for further discussion and examples of CRQ methods, see Coco & Rick, 2014; Fusaroli, Konvalinka, & Wallot, 2014). A more detailed description of parameter estimation can be found in the supplementary material (Section B).

### 3.2 Behavioral testing

First, we averaged participants’ ratings of their feelings toward the group as a whole, to calculate a group affiliation score. Then we averaged their ratings of each of their individual group members, to calculate one score for liking. For every statement in the opinion task, the distance between the mean group position and the individual participants’ positions was calculated. Averaging across all opinion items gave a score for how much a participant agreed with their group.

## 4 Results

### 4.1 Differences between synchronous and asynchronous conditions

To assess the degree to which our movement tasks produced unitary synchrony or distributed coordination, we compared SRR and DET rates between the two experimental conditions. We employed a Bayesian analysis of our results since in addition to avoiding some of the problems of null hypothesis significance testing alone (Cumming, 2014; Krushke, 2010), these analyses are able to estimate the relative strength of evidence for and against null and alternative hypotheses (Wagenmakers, Wetzels, Borsboom, & van der Maas, 2011).

**Figure 2:**
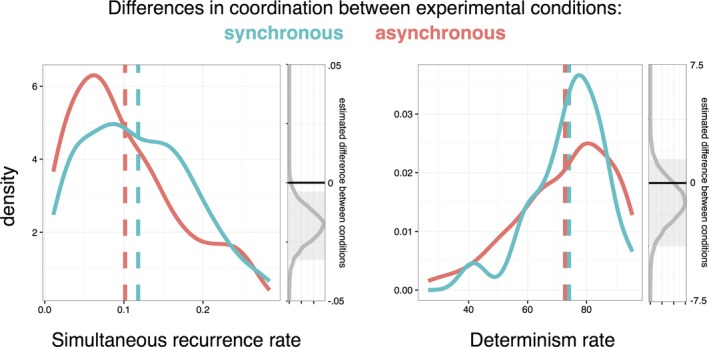
Cross‐recurrence analysis of participants’ acceleration profiles. Red and blue sold lines show density functions between movement workshop conditions and dotted lines show means. Gray lines show the distribution of estimated condition differences, and gray boxes show their 95% credibility intervals.

As shown in Fig. 2, there was a higher SRR value in the synchronous over the asynchronous condition, and a Bayesian analysis (Kruschke, 2010) of the mean difference between conditions showed that a zero difference between conditions lay outside of the 95% credibility intervals. This was not the case with DET rates, however, where there was little evidence that the difference between conditions was greater than zero. Further analyses were conducted in R using the BayesFactor package (Morey & Rouder, 2015) and default parameter values. There was a Bayes factor of 5:1 in favor of a main effect of movement condition on SRR values over the null hypothesis that there was no difference. The evidence in favor of a difference on RR rates was 0.342:1. In other words, for DET, the odds were approximately 3:1 in favor of the null hypotheses that there were no differences between experimental conditions.

**Figure 3:**
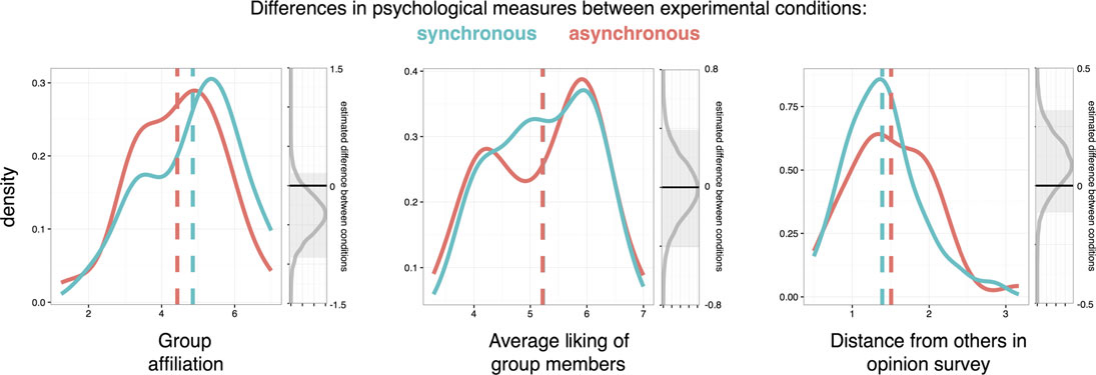
Participants’ scores on measures of affiliation, liking, and conformity. Red and blue solid lines show density functions between movement workshop conditions and dotted lines show means. Gray lines show the distribution of estimated condition differences, and gray boxes show their 95% credibility intervals.

Similar analyses were carried out across our psychological measures between synchronous and asynchronous conditions. As shown in Fig. 3, there is little evidence that the movement conditions produced any differences, according to 95% credibility intervals. Further Bayesian analysis suggested that there was no evidence for or against group affiliation scores being affected by movement condition (Bayes factor 0.60:1). There was some evidence in favor of the null hypothesis and against the hypothesis that group liking (Bayes factor 4:1) or opinion conformity (Bayes factor 3:1) was influenced by movement conditions.

In summary, as we expected, synchronous and asynchronous movement tasks produced different levels of unitary synchrony as measured by SRR, but they did not affect overall levels of movement similarity and distributed coordination as measured by DET. However, our Bayesian analysis gives evidence in favor of the null hypothesis that the movement tasks did not actually increase levels of affiliation, liking, or conformity between the groups.

### 4.2 Correlations between group motion and psychological measures

**Figure 4:**
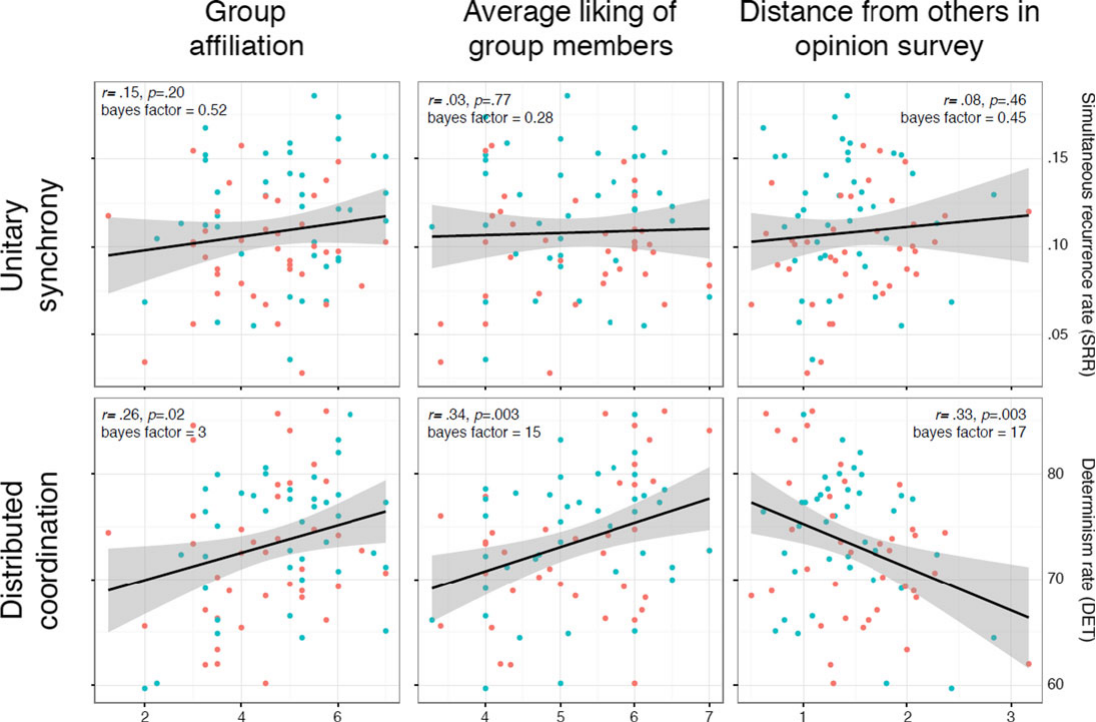
Relationship between three psychological tests and two measures of movement coordination. Black lines show regression across all individuals, with correlation coefficient, significance, and Bayes factor in support of an association.

We ran correlational analyses between the two movement measures (SRR and DET) for every individual and our three psychological measures, as shown in Fig. 4. Bayesian analysis found evidence in favor of the hypothesis that DET rates had an influence on group affiliation (Bayes factor 3:1), average liking of group members (Bayes factor 15:1), and group conformity (Bayes factor 17:1). However, for SRR, the odds were in favor of the null hypothesis, against the hypothesis that SRR had an influence on group affiliation (Bayes factor 2:1), liking (4:1), or conformity (2:1). In other words, levels of distributed coordination predicted psychological measures, but levels of unitary synchrony (which were manipulated by our task instructions) did not. Indeed, for participants across the synchronous and asynchronous conditions, both Fisher's (1925) z tests and Zou's (2007) confidence intervals found no evidence that the relationship between DET and psychological measures differed by movement workshop conditions (affiliation z = ‐.078; liking z = 0.28, opinion z = −0.78).

## 5 Discussion

Large–scale synchronous behavior—marching, dancing, or singing—can enthuse, inspire, mesmerize, or frighten, and has been part of rituals and traditions across the world over centuries (Haidt, Seder, & Kesebir, 2008; Vicary et al., 2017). Rituals bind people together (Whitehouse & Lanman, 2014), emphasizing group membership and commitment to the group (McNeill, 1995), as well as facilitating cooperation (Watson‐Jones & Legare, 2016). Despite these compelling observations, a systematic empirical analysis of many of these claims is still lacking (Whitehouse & Lanman, 2014). Specifically, what are the essential characteristics of group coordination that lead to such positive psychological effects?

We manipulated movement synchronization using a set of choreographic exercises that allowed us to identify two routes to synchrony, and we measured their social consequences. While the synchrony condition in our experiment successfully produced what we term *unitary synchrony*, it was not accompanied by increased affiliation and conformity. Instead, measures of the *distributed coordination* between pairs of participants within a group emerged as clear predictors of group bonding, irrespective of synchrony‐specific task instructions. In other words, we found evidence against the hypothesis that unitary synchrony—as manipulated in our tasks and measured by accelerometers—had any effect on how the groups felt about each other or how they interacted. In contrast, we found strong evidence that measures of distributed coordination across the groups predicted their social behavior. These correlations held equally for groups that were instructed to move together and those that were not.

Of course, we cannot discern the direction of causality in the correlation between affiliation measures and DET in group movement. It could be the case that some participants found that they liked each other more than others, and that this caused them both to couple their movements in the workshop, and to affiliate and conform to each other in the psychological tests. However, we asked participants if they knew each other prior to the workshop. The degree of familiarity did not predict DET rates, which we would expect if it reflected affiliative relationships that existed prior to the workshop. More important, however, the direction of causality does not impact our substantive claim. What is informative about our study is that affiliation either causes or is caused by patterns of distributed coordination in group movements, and not, it seems, by instructed unitary synchrony, as the current literature suggests.

So why did our instructions, guiding groups to move in synchrony or asynchrony, not influence social behavior when previous experiments showed that it did? Our study differs from previous studies in two key respects: First, we carefully avoided any references to group behavior and coordination in our task instructions, whereas previous studies often established an explicitly shared goal to synchronize (see, for example, Reddish et al., 2013). Secondly, our movement tasks lacked an external rhythmical reference signal, which would have increased *both* unitary synchrony and distributed coordination. Hence, in contrast to our experiment, group synchrony in previous studies of behavioral coordination was both instructed *and* conducted. For example, groups of participants were told to walk in time with an experimenter (Wiltermuth & Heath, 2009), were listening to the same music as they danced (Tarr et al., 2015), or performed tasks of movement or speech synchronization to the beat of a metronome (Reddish et al., 2013). The external periodic cues available to groups of participants in these studies probably enabled them to form stable behavioral patterns as they reduced the difference in eigenfrequencies of movement, facilitating entrainment (Codrons et al., 2014).

This methodological difference suggests two explanations for why our movement conditions did not predict social behaviors. The first is that when group behavior is not only instructed, but also conducted by an external signal such as a drumbeat or metronome, it will increase both unitary synchrony and distributed coordination, as we define them. Previous experiments found differences between their movement conditions because they may have conflated distributed coordination and unitary synchrony, experimentally manipulating both. The second explanation is that in our experiment, participants were instructed to move synchronously in one condition, but in the absence of a conductor found it more difficult to achieve synchronization. Under these circumstances, the synchronous groups might have interpreted any coordination difficulties as a failure of group cohesion, thereby inhibiting the emergence of pro‐social attitudes and behavior.

## Conclusion

There are many ways that a group of people can coordinate their movements, and not all of them lead to affiliation. In the absence of an external signal conducting synchrony, being instructed to move in time with each other did not predict pro‐social effects in our groups. However, independent of the synchrony instructions we gave them, a form of distributed coordination did emerge. This coordination took the form of extended movement trajectories that were echoed between participants. The degree to which this distributed coordination emerged in some groups more than others predicted their social behavior and reflected the choreography of their affiliation.

## Author contributions

All authors contributed to the overall study concept and design. M. Sperling, G. Orgs, and S. Vicary developed the movement workshop. J. von Zimmermann and D. Richardson were responsible for the development of the psychological testing session. All authors performed the testing and data collection together. S. Vicary and D. Richardson performed the data analysis. J. von Zimmermann drafted the manuscript, and D. Richardson and G. Orgs provided critical revisions. All authors approved the final version of the manuscript for submission.

## Supporting information


**Table S1**. Optimized parameters by task.
