# Supplementary material for: The Choreography of Group Affiliation
Source: Top Cogn Sci. 2018 Jan 12;10(1):80–94. doi: 10.1111/tops.12320 (PMC6092630; doi:10.1111/tops.12320)
Supplement: Supplementary file 1 — Table S1. Optimized parameters by task. [file TOPS-10-80-s001.docx]

**SUPPLEMENTARY MATERIALS**

**Section A**

**Methods**

Here are the full task instruction scripts for the circling task in the movement workshop:

| **Circle Synchronous** | **Circle Asynchronous** |
| --- | --- |
| For the first task, can we stand in a circle? Let’s start by imagining that we can see a really clear circle drawn on the floor (gesturing to indicate location of shared circle beneath group’s feet). And let’s turn to our left to face around the circle... And let’s just walk this circle. And we’re going to always try to stay equidistant, so all the gaps between us are pretty much the same size. (I step out of circle, ask participants to close the gap.) Now, let’s add the idea that, rather than the circle always staying the same size, it can begin to either get larger and expand or get smaller and shrink, while still always keeping just one clear circle in the room. So notice: how does that circle want to change its size now? Does it want to get bigger or smaller? Working to maintain that one clear circle, even while it changes in size. And let’s add the idea that whenever the one circle is getting smaller, your movement is getting slower, and whenever your circle is getting bigger, your movement is getting faster. So the smallest circle is a very slow walk and the biggest circle is very fast. How big and fast can it become and how slow and small can it become? (When small: maybe this is a good moment to turn around and walk in the other direction around the circle.) As you continue walking, let’s add another idea in: let’s now begin to allow the centre point of the circle to slide along the floor, so the whole circle is not stuck in one spot, but can slide sideways all the way across from one side of the floor to the other. In which direction does the one circle want to slide across the floor? You always maintain the one clear circle, even as it slides across the floor. How much freedom can you give the circle to keep changing in all of those ways? How far can it slide across the room? How big and fast can it be? How slow and small? Keep going just a bit longer. Take a rest, well done. | This task is about imagining a circle drawn on the floor, and walking along that circle. So let’s start with that; you imagine a complete circle drawn on the floor, you choose where you draw your circle (gesturing to indicate example location of personal circle) and you just walk along that circle, and of course feel free to do whatever you need to negotiate the space safely. Now, let’s add the idea that, rather than your movement always staying the same speed, it’s either getting faster or getting slower. It never stays the same speed for long, it’s always in the process of gradually getting faster or gradually getting slower. And the slow can be very slow and the fast can be very fast. Now let’s add the idea that your circle also begins to change its size. It’s either in the process of getting larger and expanding or getting smaller and shrinking. How does that circle want to change its size now? Does it want to get bigger or smaller? And you stay with your circle as it changes and you always maintain that clear picture of where your circle is and what it looks like. And now let’s put those two ideas together and say that whenever your circle is getting smaller, your movement is getting slower, and whenever your circle is getting bigger, your movement is getting faster. So the smallest circle is a very slow walk and the biggest circle is very fast. How big and fast can it become and how slow and small can it become? As you continue walking, let’s add another idea in: let’s now begin to allow the centre point of your circle to slide along the floor, so the whole circle is not stuck in one spot, but can slide sideways all the way across from one side of the floor to the other. And you keep clearly following your circle as it changes. In which direction does your circle want to slide across the floor? (Say once: if at any point you want to turn around and walk in the other direction around the circle, feel free.) How much freedom can you give the circle to keep changing in all of those ways? How far can it slide across the room? How big and fast can it be? How slow and small? Keep going just a bit longer. Take a rest, well done. |

**Section B**

**Data Processing**

The calculation of cross recurrence on continuous data series requires the estimation of three critical parameters, namely the threshold or Radius of values to be considered as recurrent (i.e. how far apart two values can be and still be considered synchronous), the Delay to be incorporated (i.e. how many data points are used to estimate synchrony) and the number of Embedding Dimensions to be used (i.e. how many points within the delay are used to estimate the ‘recurrence’ of the two series). Since each task had particular movement characteristics, we would expect these parameters to differ by task. We used the ‘optimize parameters’ function to estimates these values empirically for every unique pair of participants within each group, for each task. Then for each task we took the average of all pairwise estimates to set the parameters for the recurrence analysis (see Table 1).

**Table 1**. Optimized Parameters by Task

|  | Circles Task | | Falling Task | | Swinging Task | |
| --- | --- | --- | --- | --- | --- | --- |
|  | M | SD | M | SD | M | SD |
| Radius | **0.278** | 0.152 | **0.081** | 0.084 | **0.303** | 0.194 |
|  |  |  |  |  |  |  |
| Embedding | **7.619** | 2.807 | **3.126** | 3.002 | **7.303** | 4.096 |
|  |  |  |  |  |  |  |
| Delay | **130.946** | 66.174 | **115.622** | 80.165 | **123.554** | 64.680 |
